# Use of Hydrogen Peroxide Vapour for Microbiological Disinfection in Hospital Environments: A Review

**DOI:** 10.3390/bioengineering11030205

**Published:** 2024-02-22

**Authors:** Aaqib Ayub, Yuen Ki Cheong, Jesus Calvo Castro, Oliver Cumberlege, Andreas Chrysanthou

**Affiliations:** 1 School of Physics, Engineering and Computer Science, University of Hertfordshire, Hatfield AL10 9AB, UK; a.ayub4@herts.ac.uk (A.A.); a.chrysanthou@herts.ac.uk (A.C.); 2 School of Life and Medical Sciences, University of Hertfordshire, Hatfield AL10 9AB, UK; j.calvo-castro@herts.ac.uk; 3 Ecolab (Formerly Bioquell UK), Andover SP10 3TS, UK

**Keywords:** high-level disinfection, decontamination, hospital-acquired infections, biocides, hydrogen peroxide vapour, SARS-CoV-2, N95 respirators, FFP2 masks, COVID-19

## Abstract

Disinfection of nosocomial pathogens in hospitals is crucial to combat healthcare-acquired infections, which can be acquired by patients, visitors and healthcare workers. However, the presence of a wide range of pathogens and biofilms, combined with the indiscriminate use of antibiotics, presents infection control teams in healthcare facilities with ongoing challenges in the selection of biocides and application methods. This necessitates the development of biocides and innovative disinfection methods that overcome the shortcomings of conventional methods. This comprehensive review finds the use of hydrogen peroxide vapour to be a superior alternative to conventional methods. Motivated by observations in previous studies, herein, we provide a comprehensive overview on the utilisation of hydrogen peroxide vapour as a superior high-level disinfection alternative in hospital settings. This review finds hydrogen peroxide vapour to be very close to an ideal disinfectant due to its proven efficacy against a wide range of microorganisms, safety to use, lack of toxicity concerns and good material compatibility. The superiority of hydrogen peroxide vapour was recently demonstrated in the case of decontamination of N95/FFP2 masks for reuse to address the critical shortage caused by the severe acute respiratory syndrome coronavirus 2 (SARS-CoV-2) during the COVID-19 pandemic. Despite the significant number of studies demonstrating antimicrobial activity, there remains a need to critically understand the mechanism of action by performing studies that simultaneously measure damage to all bacterial cell components and assess the correlation of this damage with a reduction in viable cell count. This can lead to improvement in antimicrobial efficacy and foster the development of superior approaches.

## 1. Introduction

Disinfection is described as a process that eliminates many or all pathogenic microorganisms on inanimate objects, with the exception of bacterial endospores [1]. Disinfection is usually carried out by chemical or physical means [1]. Among other settings, disinfection is of utmost importance in hospital environments due to pathogens living on hospital surfaces being the direct cause for hospital-acquired infections (HAIs). HAIs, also referred to as healthcare-acquired infections [2], are infections that are not present or are not incubating at the time of hospital admission [3]; these can be acquired by patients, visitors and healthcare staff. HAIs also include infections acquired in healthcare settings outside of hospitals; such settings include ambulatory care, care homes and family clinics [4]. These infections can appear 48 h after hospital admission or within 30 days of receiving care [3]. HAIs have been one of the major causes of increases in deaths among patients receiving care in healthcare settings [2]. HAIs are a major risk not only to patient health but also to occupational care staff and hospital visitors. The number of patients acquiring HAIs when in hospital care within the National Health Service in the United Kingdom is estimated to be 300,000 per annum [5]. Infections acquired in hospitals have an adverse effect on patient outcomes (increase in the duration of hospital stay and exposure to new infections) and increase the mortality rate and costs associated with patient care [2,6]. In the United States alone, HAIs are estimated to impact two million patients each year, resulting in around 90,000 deaths per year and an estimated direct cost of USD 28 to USD 45 billion [6]. An exponential growth in the number of HAIs has been observed since the 1980s mainly due to the emergence of multidrug-resistant bacteria [7,8,9]. The indiscriminate use of antibiotics is a major contributing factor to this as it has led to some bacteria acquiring drug resistance. The increasing number of HAIs is a matter of serious concern as they can lead to severe illnesses, deaths and high healthcare costs.

Pathogens causing HAIs can spread through the touch of infected surfaces. Some studies have shown that pathogens can infect and survive on an inanimate surface from a period of a few hours to years [10,11]. For example, *Escherichia coli* (*E. coli*) can survive on dry inanimate surfaces from 1.5 h to 16 months, while *Clostridium difficile* (*C. difficile*) can survive on dry inanimate surfaces and hospital floors for a period of up to 5 months [10,12]. *E. coli* spreads through the ingestion of contaminated food, milk or water, as well as through person-to-person transmission, leading to blood and urinary tract infections [13]. *C. difficile* spreads through extensive surface contamination and causes diarrhoea and colitis [12,14]. The ability of these clinically relevant nosocomial pathogens to survive on hospital surfaces has led to the need for disinfection or, in simple terms, to the need to kill these disease-causing microorganisms. Indeed, clinical studies have shown that poor environmental hygiene can lead to the transmission of pathogens [15]. Pathogens living on surfaces or on shared and non-shared equipment in hospitals can lead to hand contamination (upon contact) and to further transmission to equipment, patients and high-touch surfaces [16]. Pathogen transmission occurs via patients and healthcare staff by coming into contact with high-touch surfaces, such as door handles, beds, taps and telephone receivers [16]. Further transmission can also occur by commonly shared clinical equipment like stethoscopes, which come into contact with intact skin. In addition, during the procedures of urinary catheterisation and gastrointestinal endoscopy, medical instruments come into contact with sterile or mucous tissues, and thus, both of these can lead to increasing infections. This is of particular concern because 25% of hospitalised patients in the United States need catheterisation, while 10 million gastrointestinal endoscopies are carried out every year [17]. Records show that 30% of patients receiving urinary catheterisation show systemic symptoms that relate to catheter-associated urinary tract infections caused by HAIs [18]. Urinary tract infections (UTIs) are the second most common type of HAIs in the United Kingdom, accounting for 17.2% of all HAIs, while pneumonia and other respiratory tract infections account for 22.8% of the total [5].

A critical evaluation of the available literature on HAIs highlights the need for effective and efficient disinfection methods and biocides, followed by the correct use of disinfection techniques/methods. The literature contains a significant number of studies that document the efficacy of disinfectants and their antimicrobial action. However, not all biocides are effective against all types of pathogens. Furthermore, not all disinfection methods are seen to be effective [19,20,21]; in fact, in one investigation, ready-to-use antibacterial wipes were observed to act as pathogen spreaders instead of eradicating pathogens [22]. Hence, both the selection of a biocide and the method of disinfection are important in determining the correct disinfection strategy. New and efficient approaches are, therefore, required, particularly in the case of some multidrug-resistant organisms that are found on hospital surfaces in communities known as biofilms. Biofilms act as a reservoir of pathogens, and their intrinsic properties make them resilient against disinfectants. Biofilms are multicellular communities held together by a self-produced extracellular matrix [23]. Such bacterial biofilms have been shown to be 1500 times more resistant to biocides than planktonic bacteria growing in liquid cultures [24]. Along these lines, Vickery et al. [25] carried out a study on bacterial biofilms by disinfecting clinical samples using chlorine-based disinfectants and noticed the presence of biofilms for periods of 12 months even after routine cleaning. These observations pertain to the consideration that it is not just the types of organisms present but also the form they are in (e.g., in biofilms) that can impact the efficacy of a biocide. In addition to antimicrobial activity, infection control teams must assess a biocide for selection based on its safety to users and the environment and consider the compatibility of the biocide with the treated materials. Among the large variety of biocides used and reported to date, hydrogen peroxide (H_2_O_2_) presents an attractive option due to its demonstrated wide-range sterilant activity, its surface material compatibility and its safety to end users [4].

This review critically discusses the use of H_2_O_2_ as a biocide in hospital environments, its antimicrobial activity against clinically relevant pathogens, and its mechanism of action, as well as its recent use for the decontamination of N95/FFP2 face masks for reuse to address the critical shortage caused by the occurrence of severe acute respiratory syndrome coronavirus 2 (SARS-CoV-2).

## 2. Hydrogen Peroxide Vapour as a Biocide

Hydrogen peroxide is used as a disinfectant/sterilant by being applied directly in the form of an aqueous solution at a concentration ranging from 3 to 9% (*w*/*w*) [26]; it is formulated with different chemicals in water or gas, in an aerosolised form or in a vapour form [27]. The use of H_2_O_2_ as a biocide is found in multiple industries, including the food and beverage sector, agriculture, hospitals, the pharmaceutical and cosmetic sector, the water supply industry, and the public and commercial disinfection industry [4,28]. Its use in the food and beverage sector in a liquid form is targeted for disinfection and sterilisation of food contact surfaces that are used for milk and juice storage and for the preservation of water, milk and juices [28]. The use of H_2_O_2_ in the pharmaceutical and cosmetic industry takes the form of liquid formulations at concentrations ranging from 3 to 9% (*v*/*v*) in various products including wound applicants, oral disinfection in dentistry, contact lens disinfection, and as a preservative in cosmetics [29,30,31]. Furthermore, higher concentrations of hydrogen peroxide solutions are used in the manufacturing of foam rubber, organic compounds, rocket fuel, and bleach for paper and textiles [31]. Examples of use in commercial sterilisation and in the water industry include industrial effluent treatment, algae control in water and wastewater deodorisation. The use of H_2_O_2_ in vapour form is found widely in the healthcare sector for disinfection and sterilisation [4]. In addition to its use against bacteria, hydrogen peroxide in vapour form is shown to be effective against a variety of organisms including certain types of hard-to-kill nematode worms and prions, thus finding use in animal husbandry as well [27,32]. This widespread use of H_2_O_2_ in multiple industries is due to it being considered an “ideal” biocide depending on how it is used [4]. An “ideal” biocide as defined by McDonnell [27] as one that must be safe to use, easy to store, easy to apply and have a long-lasting effect, as well as being environmentally friendly and being chemically compatible with the surface it is applied on. An assessment of hydrogen peroxide vapour (HPV) against the attributes of an ideal biocide can be outlined as follows:

(i) **Efficacy**: A significant number of in vitro and in vivo studies have demonstrated the efficiency of H_2_O_2_, both in liquid and vapour phases, against organisms ranging from highly resistant bacterial endospores to enveloped viruses [4,19,32,33,34,35]. According to these studies, antimicrobial activities depend on the concentration of H_2_O_2_, the exposure time and the method of application.
(ii) **Safety**: Hydrogen peroxide is applied to the skin for wound disinfection and used in acne products in liquid form at a low concentration of less than 3% *w*/*w*; this concentration level is considered very safe for use on human skin [30,36]. However, with an increase in concentration, a decreased tissue compatibility has been reported [30,36]. The safety of H_2_O_2_ is entirely dependent on how it is used. Owing to the absence (or to the low toxicity effects), H_2_O_2_ is seen as an excellent option for replacing more toxic chemicals like formaldehyde, which is known to be carcinogenic, and ethylene oxide, which has high toxicity and carcinogenicity concerns [37,38,39]. A major advantage of modern hydrogen peroxide vapour systems is that they can be easily set up and operated remotely, thus eliminating contact with the operator and reducing risk. The permissible exposure limit weighted over 8 h by the OSHA (Occupational Safety and Health Administration) in the United States is 1 ppm, whereas an immediate danger to life or health is posed at 75 ppm [40].
(iii) **Environmental impact**: The environmental impact of hydrogen peroxide is entirely dependent on how it is used. HPV slowly decomposes into water and oxygen and, because of this, it is considered safe for the environment [41]. As a result, no harmful residues are left on surfaces. The relatively unstable peroxide bond leads to its natural decomposition.
(iv) **Ease of use**: Factors that impact the ease of use of H_2_O_2_ are its concentration and method of application. For example, hydrogen peroxide is highly effective when used in vapour form as it can easily reach crevices and other hard-to-reach areas. This can also be ideal for large-area decontamination as multiple machines can be used at the same time. Modern, no-touch HPV systems reduce the number of labour hours when compared with traditional decontamination methods, leading to a reduction in labour costs.
(v) **Stability**: Hydrogen peroxide is stable in water and other formulations, depending on its purity and storage conditions. It is important that hydrogen peroxide is stored under conditions recommended by the manufacturer. Dissociation of hydrogen peroxide can take place if stored incorrectly. This will reduce the concentration of hydrogen peroxide in the solution and will have an impact on its antimicrobial efficacy.
(vi) **Compatibility with surface materials**: Hydrogen peroxide can be safe to surfaces, depending on how it is used. Being an oxidising agent, it can oxidise certain metallic and plastic surfaces when used in higher concentrations in liquid form [4]. However, these effects can be prevented when H_2_O_2_ is used in vapour form, which is considered to be gentle to surfaces and electrical equipment that are key parts of hospital environments. Boyce et al. [42] studied the impact of microcondensation HPV room decontamination on hospital physiological monitors over an 8-year period and observed that there was no increase in maintenance service calls; in fact, a rather unexplained decrease in maintenance was apparent. Furthermore, a recent study by Sher and Mulder [43] on the use of vapour-phase and aerosolised hydrogen peroxide for disinfection of dental surgery areas found no damage to any surface in these surgery areas. The effect of HPV on three metallic materials was characterised by Gale et al. [44], and no systematic effects were seen on the tensile strength or post-HPV-treated corrosion resistance of the alloys tested. Microstructural changes were seen to be confined to the areas adjacent to the exposed surface and were considered to be relatively small [44].

Commercial disinfection systems commonly generate hydrogen peroxide vapour by controlled heating of a 35% *w*/*w* aqueous solution [45]. The solution is continuously refilled in the evaporator as the phase change from liquid to vapour takes place [46]. Commercial systems can use a hot plate to flash evaporate a 35% (*w*/*w*) hydrogen peroxide solution [47]. The resulting vapour is continuously fed to the room, and some researchers suggest that microcondensation can be formed at ~3 μm thickness on surfaces [46]. The hydrogen peroxide vapour can then be made to decompose into water vapour and oxygen upon catalysis by an active aeration system [48,49]. A number of studies [50,51] have shown that hydrogen peroxide in vapour form, even at low concentrations, is highly efficient when compared to liquid hydrogen peroxide. This has been attributed to the higher level of interaction between macromolecules (molecules considerably larger than an ordinary molecule that contain a larger number of atoms), where greater oxidation has been observed when the peroxide is in vapour form [51]. It is important to recognise that there are various commercially available hydrogen peroxide vapour systems, and these can use significantly different methods [46]. Due to the fundamental differences in the delivery methods used by these processes, it is well known that they yield noticeably different disinfection results [52,53]. The term vaporised hydrogen peroxide^®^ (VHP^®^) refers to a process that lowers the relative humidity (RH) of the room being disinfected before adding peroxide to avoid reaching the dew point and condensation, and then the process is regulated to a predetermined concentration by removing vapour and adjusting the hydrogen peroxide injection rate to avoid reaching the dew point. In contrast, HPV is the term used for the process whereby vapour is purposefully delivered to reach the dew point and condensation by recirculating peroxide and adding more vapour [45]. Although VHP and HPV have previously been used indistinguishably in a study on decontamination of N95 respirators [53], one must note the difference between the two. Additionally, the neutral term, vapour-phase hydrogen peroxide (VPHP), is employed to refer to both the HPV and VHP procedures, as well as other similar processes. The ISO term for all these systems is vaporised hydrogen peroxide (VH_2_O_2_) [54].

## 3. Application of Hydrogen Peroxide Vapour against Clinically Relevant Pathogens

The decontamination of healthcare environments by using hydrogen peroxide vapour has been extensively studied because of its excellent antimicrobial efficacy. HPV systems have demonstrated antimicrobial efficacy against pathogens ranging from highly resistant bacterial endospores to the least resistant enveloped viruses, as classified by Spaulding [55]. A significant number of in vitro studies have demonstrated the microbial efficacy of HPV against frequently reported clinically relevant pathogens. HPV systems have achieved a greater than 6 log_10_ (greater than 99.9999%) reduction in pathogens, as validated by their efficacy against *Geobacillus stearothermophilus* ATCC 7953 biological indicator spores [19,33]. A greater than 6 log_10_ reduction has also been achieved against other clinically relevant pathogens, such as *C. difficle*, *methicillin-resistant Staphylococcus aureus* (*MRSA*), *Vancomycin-resistant enterococci* (*VRE*), *norovirus surrogates* and *Acinetobacter baumannii* (*A. baumannii*) [48,52,56,57,58,59,60]. The use of hydrogen peroxide vapour has been found to be effective in removing environmental reservoirs of *C. difficle* [61], *MRSA, methicillin-susceptible Staphylococcus aureus* (*MSSA*) [19,62], *multi-resistant Gram-negative bacteria* [33,63] and others [34].

These studies have demonstrated both antimicrobial efficacy and repeatability, which provides confidence in the use of HPV for decontamination. Furthermore, commercially available automated HPV disinfection systems can deliver concentrations of the fumigant required to achieve the criteria stated in the EN17272 standard [64]. The EN17272 is a European standard for determining the disinfectant activity of airborne room disinfection by automated processes and covers vegetative bacteria, mycobacteria, bacterial spores, yeasts, fungal spores, viruses and bacteriophages [65].

The use of HPV as a decontaminant has demonstrated significant potential in combating multidrug-resistant organisms (MDROs) in healthcare settings. HPV as a decontaminant provides an effective and practical method for reducing the environmental load of microorganisms resistant to several antibiotics, such as MRSA and VRE. Studies [66,67] have shown that HPV may significantly decrease the number of these bacteria on various hospital surfaces, thereby lowering the risk of healthcare-associated illnesses (HAIs). Khandelwal et al. [66] conducted a study in a critical care setting and found that hybrid hydrogen peroxide fogging could lower the bacterial counts on crucial surfaces, implying that this strategy is more effective than regular cleaning practices and ultraviolet light use in removing MDROs. Furthermore, a comprehensive review and meta-analysis by Marra et al. [67] confirmed the efficacy of no-touch disinfection technologies like HPV, revealing a statistically significant reduction in infections caused by particular MDROs such as *C. difficile* and VRE. These findings highlight the necessity of implementing modern disinfection technologies, such as HPV, into infection control regimens to improve patient safety and combat the spread of resistant pathogens.

HPV systems are known to have a positive impact on the reduction in infections in clinical settings, as demonstrated by three major studies [68,69,70]. A quasi-experimental study involving a 900-bed community hospital was conducted by Manian et al. [68]. Enhanced cleaning was performed using bleach, followed by HPV disinfection of rooms vacated by patients with *C. difficile*-associated diarrhoea. The rate of *C. difficile*-associated diarrhoea infection dropped hospital-wide by 37%, with the authors being able to demonstrate the safe use of HPV in a large hospital. Similar results were observed by Boyce et al. [69], who conducted a before-and-after intervention study in a hospital affected by an epidemic strain of *C. difficile*. HPV disinfection was reported to be efficacious in removing *C. difficile* from contaminated surfaces, and the incidence of *C. difficile*-associated infections post-HPV intervention was reduced to 0.88 cases/1000 patients from 1.89 cases/1000 patients pre-HPV intervention [69]. Furthermore, Passaretti et al. [70] demonstrated that the risk of a patient acquiring an infection (with multidrug-resistant organisms) was 64% less likely after a room has been sterilised with HPV compared to rooms cleaned using “traditional” processes. These studies demonstrated a reduction in the incidence of new infections and a lower risk to patients.

## 4. Effects on Bacteria

Some of the initial studies used HPV as a decontaminant targeting *E. coli*, a clinically relevant pathogen that is the most frequently reported pathogen and accounts for 17.5% of the total pathogens reported from over 5626 healthcare facilities in the United States for the period of 2015–2017 [71]. Back et al. [71] performed a study subjecting three strains of *E. coli* inoculated on lettuce to 10% HPV for 10 min. The authors [71] reported that the treatment led to a reduction level of 3.15 log_10_ CFU/g (colony-forming unit per gram) for *E*. *Coli* O157:H7. In another study by Benga et al. [72], similar results were reported after treatment with HPV, demonstrating a complete disinfection of *E. coli* and other bacterial species (inoculated on bedding pieces housed in a mouse facility) in the presence of water and bovine serum albumin (BSA) solutions.

The effectiveness of HPV was further demonstrated in a study by Otter et al. [48], who investigated the surface survival of commonly found spores and vegetative bacteria such as *Staphylococcus aureus* (*S. aureus*), the second most commonly found nosocomial pathogen in healthcare settings [73]. While most vegetative bacteria and spores with inocula of 6 log_10_ CFU to 7 log_10_ CFU survived on surfaces for more than 5 weeks in a 100 m^3^ test room, they were inactivated within 90 min of exposure to HPV, even in the presence of 0.3% bovine serum albumin that was used to simulate biological soiling. In another investigation, Lemmen et al. [74] evaluated the performance of HPV in the disinfection of organisms such as *MRSA*, *vancomycin-resistant Enterococcus* (*VRE*) and *Acinetobacter baumannii* (*A. baumannii*), which were located on porous and non-porous surfaces using cotton and stainless steel as carriers in an operating room. The experiment was repeated three times and, at each instance, no pathogens were found on either porous or non-porous carriers after being subjected to automated HPV disinfection [74]. *Klebsiella pneumoniae* (*K. pneumoniae*), the third most common nosocomial organism that is also found in healthcare settings [73], is known to cause urinary tract infections, pneumonia, septicemias and soft tissue infections [75] and can survive on inanimate surfaces from 2 h to 30 months [10]. The work of Ali et al. [76] compared the efficacy of two different HPV systems using significantly different hydrogen peroxide concentrations in single isolation rooms, while using an aerobically inoculated sterile broth of centrifuged *K. pneumoniae* suspended in 0.03% BSA (*w*/*v*) and 10% BSA (*w*/*v*) to simulate low and heavy soil loadings. It was shown that enhanced cleaning with HPV reduced the risk of cross-contamination by killing the left-over surface contamination that was present after manual terminal cleaning. A study [72] was also conducted on *Klebsiella oxytoca* (*K. oxytoca*), which is known to cause HAIs in adults and has developed resistance to commonly used antibiotics [77]. The application of HPV by Benga et al. [72] on bacteria of laboratory animal origin showed that *K. oxytoca* and other bacterial species were readily disinfected upon being treated. Similar disinfection results were observed when BSA was smeared on smooth surfaces to simulate soiling [72].

A study conducted by Watson et al. [78] on the effect of HPV on *Pseudomonas aeruginosa* (*P. aeruginosa*) used bacterial biofilms generated by a drip flow reactor. These biofilm samples were subjected to HPV treatment in an enclosure using a commercial vapour generator. The results after 100 min of exposure to HPV showed a reduction greater than 6 log_10_ in the enclosed room-based scenario. The microscopic results after the HPV treatment revealed a noticeable impact on the disruption of microcolony formation. To further compare HPV decontamination with conventional terminal cleaning, Otter et al. [79] compared hydrogen peroxide vapour decontamination with conventional terminal cleaning. Their work involved reservoirs of multidrug-resistant Gram-negative rods (MDR-GNRs), such as *Enterobacter cloacae*, in a 1389 m^3^ intensive care unit (ICU) room using samples from different locations and placing 40 Tyvek-pouched 6 log_10_ *Geobacillus stearothermophilus* ATCC 7953 biological indicators along the periphery. The results suggested that HPV decontamination was more efficacious than conventional terminal cleaning. The removal of environmental reservoirs of MDR-GNRs could also have stopped the cycle of transmission of these organisms.

The results of these studies demonstrated the effectiveness of HPV as a disinfectant and biocide against the most common bacteria. Impressive results were generally achieved quickly within about 100 min (dependent on room size) of exposure to HPV. This is interesting as such treatment can potentially be applied in hospitals, leading to a reduction in operational disturbance. Examples of studies demonstrating the efficacy of hydrogen peroxide vapour against clinically relevant bacteria that are commonly found in hospitals can be found in Table 1.

## 5. Effects on Fungi

*Candida* spp., a well-known fungus that can cause HAIs in the gastrointestinal tract, the vagina and the oral cavity [89], is known to survive for a period from 1 to 120 days on dry inanimate surfaces [10]. Due to its clinical relevance, an in situ study was carried out on samples that were purposefully collected during a *Candida auris* (*C. auris*) outbreak at the Royal Brompton Hospital in London, with the infection control methods being documented [90]. The authors [90] demonstrated a successful outcome through the use of a new infection control method that applied a high-strength chlorine-based agent, followed by hydrogen peroxide vaporisation. Examples of studies demonstrating the efficacy of hydrogen peroxide vapour against clinically relevant fungi that are commonly found in hospitals can be found in Table 2.

## 6. Effects on Viruses

Viruses spread via respiratory droplets or by direct contact [92], as well as via aerosolisation after sweeping and via fomites [10], and account for 90% of all respiratory diseases. According to an annual report published by Public Health England on the surveillance of influenza and other respiratory viruses in the UK for the winter of 2018–2019, 26,408 deaths were attributed to influenza viruses. A total of 84.2% of those deaths occurred in the age group of 65+ years. The transmission of influenza results in a high impact on health services in terms of an increase in the number of hospitalisations and ICU admissions and a significantly higher mortality rate [92]. Some of the early studies explored the effect of HPV on common viruses including the *Influenza virus*, *Avian Influenza virus*, *Influenza A* (*H1N1*) and *Swine Influenza Virus* (*H3N2*) [35]. Heckert et al. [35] studied the effect of HPV on the decontamination of equipment and inanimate materials that were potentially contaminated with a variety of animal and mammalian viral agents belonging to the *Orthomyxoviridae*, *Reoviridae*, *Flaviviridae*, *Paramyxoviridae*, *Herpesviridae*, *Picornaviridae*, *Caliciviridae* and *Rhabdoviridae* virus families. The authors reported a high efficacy of HPV; for all the viruses tested under all conditions (except one), the viral titre was reduced to 0 embryo-lethal doses for all avian viruses and to less than 10 tissue culture-infective doses for mammalian viruses [35]. Furthermore, the authors recommended the use of HPV for decontamination of potentially virus-contaminated objects in biocontainment level III laboratories that handle exotic animal disease viruses. Similar effects were reported by Rudnick et al. [93] in a study wherein HPV was applied to influenza viruses, which were deposited on stainless-steel surface coupons and exposed to HPV at different concentrations ranging from 10 to 90 ppm. It was reported that a 99% inactivation rate of influenza viruses was achieved after only 2.5 min of exposure to the lowest studied concentration of 10 ppm. Even better results were achieved at higher HPV concentrations. This outcome was further supported by a different study by Goyal et al. [94], wherein SARS-CoV-2 surrogates, such as *feline calicivirus*, *human adenovirus type 1*, *transmissible gastroenteritis coronavirus of pigs and influenza viruses,* were subjected to HPV exposure, and no viable viruses were observed, with the study achieving greater than 4 log_10_ reduction post treatment. Such results provide confidence in the efficacy of the use of HPV for surface inactivation of viruses. Examples of studies demonstrating the efficacy of hydrogen peroxide vapour against clinically relevant viruses commonly found in hospitals can be found in Table 3.

## 7. Mechanism of Biocidal Action

Hydrogen peroxide in liquid and gaseous forms has been shown to provide excellent antimicrobial activities against a broad spectrum of organisms. However, there is a lack of knowledge of the mechanism underlying its biocidal action; in spite of its demonstrated effectiveness in destroying infectious microorganisms, there remains a need to critically understand its mechanism by performing studies that simultaneously measure damage to all bacterial cell components and assess the correlation of this damage with a reduction in viable cell count [51]. The main mechanism leading to decontamination through the use of hydrogen peroxide has been thought to be via the deactivation of microorganisms through the oxidation of macromolecules that form viral and cellular structure/function, such as lipids, carbohydrates, proteins and nucleic acids [4,27]. However, in a study by Linley et al. [95] on the mechanism of cytotoxicity and genotoxicity of H_2_O_2_, it was proposed that the mechanism is due to localised formation of short-lived hydroxyl radicals through the intracellular reaction between Fe^2+^ ions and H_2_O_2_ (known as the Fenton’s reaction). Evidence for the Fenton’s reaction leading to the biocidal action of H_2_O_2_ on bacterial cells was sought by Repine et al. [96], who grew *S. aureus* bacteria in a nutrient broth with increased concentrations of iron. This approach effectively increased the iron content in *S. aureus* cells, and this was associated with a significant enhancement in the killing of bacterial cells when they were exposed to H_2_O_2_. The destruction of the cell walls of bacteria is dependent on the overall extent of peroxide-induced damage and on the effect on target cells, which have the ability to repair DNA damage. This implies that bacterial strains that are exposed to H_2_O_2_ have a reduced ability to repair DNA damage and are, therefore, more susceptible to be killed from exposure to H_2_O_2_ [96]. Since viruses have no repair mechanisms, McDonell [4] has suggested that excessive damage to viral nucleic acids should, therefore, be considered important in the overall virucidal effect. However, there is no evidence to support this. Indirect evidence of DNA damage in *E. coli* following exposure to H_2_O_2_ was provided by Imlay and Linn [97], who also proposed two kinetically distinguishable modes of killing of bacteria. The killing of bacterial cells at lower H_2_O_2_ concentrations was referred to as mode one and was reported to take place by means of DNA damage. Mode-one killing was observed to be maximal at concentrations between 1 and 2 mM of H_2_O_2_ [97]. Exposure to H_2_O_2_ was observed to lead to damage in a dose-dependent manner; this damage could undergo repair during a growth lag, but while cell growth occurred, there was no evidence of septation. The failure to successfully complete the repair of cells would lead to mode-two killing, which was evident at higher H_2_O_2_ concentrations. The authors [97] thought that mode-one killing was probably internal, while mode-two killing could be external. If this were indeed the case, mode-one killing would be expected to be diffusion-controlled. However, an earlier investigation by Schwartz et al. [98] had suggested otherwise.

Brandi et al. [99] noted a similar pattern of bimodal killing in their study on the effect of HPV on *E. coli*. These authors suggested that cell membrane damage leading to a reduction in cell volume is the major component of mode-two killing, whereas no such effect was seen in mode-one killing. This observation actually strengthens the proposal that the biocidal mechanism upon exposure to H_2_O_2_ is due to the Fenton’s reaction via mode-two killing and is dependent on the presence of hydroxyl radicals, unlike mode-one killing. Furthermore, it is important to note that the oxidation–reduction potential (ORP) of hydrogen peroxide in a solution plays a crucial role in the mechanism of antimicrobial action. The extent and the rate of the Fenton’s reaction in a solution will be directly affected by the ORP of the solution. A higher ORP indicates a more oxidising environment, implying a greater tendency for H_2_O_2_ to donate electrons and form hydroxyl radicals; hence, a more efficient and potent antimicrobial action could be expected.

**Figure 1:**
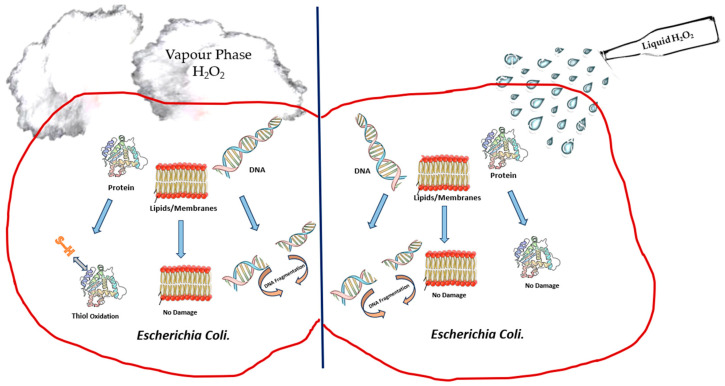
Depiction of damage to cell components of *E. coli* upon treatment with liquid-phase hydrogen peroxide and vapour-phase hydrogen peroxide.

According to Finnegan et al. [51], vaporised hydrogen peroxide interacted differently against amino acids when compared to liquid hydrogen peroxide. These authors [51] observed that liquid hydrogen peroxide at different concentrations was able to oxidise amino acids like cysteine, methionine, lysine, histidine and glycine, whereas vaporised hydrogen peroxide was unable to oxidise amino acids [51]. However, vapour-phase hydrogen peroxide was able to degrade aldolase and BSA completely, whereas no impact was observed when hydrogen peroxide was used in the liquid phase. The damage to various macromolecular cell targets upon the treatment of *E. coli* with liquid- and vapour-phase hydrogen peroxide, as studied by Linley [100], is depicted in Figure 1. Similar results showing that vapour-phase hydrogen peroxide was able to degrade protein oxidatively in comparison to liquid-phase hydrogen peroxide were also reported by McDonnell [101] in his studies on the neutralisation of bacterial protein toxins. These studies serve to highlight the difference in efficacy between vapour- and liquid-phase hydrogen peroxide. The difficulty with most of these studies in understanding the mechanism of killing of bacteria through the use of hydrogen peroxide vapour is that entire cells are exposed to hydrogen peroxide, and this results in a variety of direct and indirect effects as the causes for cell death.

There is still a need for further work to be carried out to improve the current understanding of the exact killing mechanism of vapour-phase hydrogen peroxide. Earlier studies from the 1990s, such as that of Klapes and Vesley [39], considered the application of vapour-phase hydrogen peroxide as a sterilant to be “clearly still in its infancy” due to the lack of understanding of the mechanism of action and the factors influencing its effects. Almost twenty years later, Hall et al. [60], in their study on using hydrogen peroxide vapour to deactivate *Mycobacterium tuberculosis*, stated that “the exact mechanism of action of HPV remains to be fully elucidated”.

## 8. Hydrogen Peroxide Vapour as a Biocide for Reuse of N95/FFP2 Face Masks during the COVID-19 Pandemic

The current pandemic caused by the novel severe acute respiratory syndrome coronavirus 2 (SARS-CoV-2), epi-centred in Hubei province in the People’s Republic of China, has been overwhelming healthcare systems and negatively impacting world economies [102,103]. An extreme shortage of critical N95/FFP2 masks used as Personal Protective Equipment (PPE) in healthcare settings occurred at the beginning of the COVID-19 pandemic. N95/FFP3 masks are arguably the most critical part of PPE for healthcare workers due to the aerosol transmission of SARS-CoV-2. Even though the supply of N95/FFP2 masks has improved with time, new and rapidly spreading variants of SARS-CoV-2 still pose a threat of critical shortage due to the increase in demand and the fast depletion of existing supply lines. To address such shortage, decontamination of face masks for reuse was investigated as a viable option. As HPV is widely used for surface disinfection in hospital environments and is effective against SARS-CoV-2 surrogates on surfaces [94], it was, therefore, considered as a method of inactivation. Since the beginning of the COVID-19 pandemic, numerous in vitro studies have been published on the use of HPV for decontamination of N95/FFP2 face masks [104,105,106,107]. Wigginton et al. [104] studied the decontamination of 3M-1860 N95 masks, including the filtration efficiency and integrity, using a HPV whole-room decontamination system and other methods. A 1.5 log_10_ to greater than 4 log_10_ inactivation of the tested viruses was measured when using the HPV system. The integrity of the face seal and the filtration efficiency were observed to not be affected after five cycles. Decontamination with other methods, like the use of ethylene oxide, raised toxicity concerns, while the hydrogen peroxide gas plasma decontamination method led to a decrease in the filtration efficiency [104]. Further studies by Oral et al. [105], who used a HPV system, found high-level inactivation of viruses and biological indicators after one cycle and no evidence of detrimental effects on the fit for use and the filtration efficiency. The authors of [104] are conducting further testing to determine the number of times a mask can be reprocessed. However, the HPV systems by the Battelle Memorial Institute, with emergency use authorisation from the United States Food and Drug Administration [108] for decontamination of N95 masks at the onset of the COVID-19 pandemic, were approved to be used for 20 cycles. Similar results were observed in a study by Kumar et al. [106], wherein four different N95 mask model types were tested for decontamination with HPV and other methods. Full inactivation of SARS-CoV-2 or *Vesicular stomatitis* by HPV was observed, with no loss in the function and structural integrity of these masks up to a minimum of ten cycles. In another investigation, Kenney et al. [107] further demonstrated the effectiveness of HPV whole-room systems for decontamination of N95 masks inoculated with bacteriophages. The authors found a high virucidal activity post HPV treatment of N95 masks, with acceptable limits for filtration efficiency (>99%), up to three cycles. The filtration efficiency was seen to fall below 95% after five cycles, and hence, the authors [107] recommended that the reuse of these masks after decontamination should be limited to three cycles only.

A study by Schwartz et al. [109] described the process used and demonstrated that HPV was an efficacious method for the decontamination of N95/FFP2 face masks in terms of both its ability to kill pathogens and preserve the structural integrity and functionality of these masks. Perkins et al. [110] highlighted the low toxicity of the HPV processes. From the results of these investigations, the use of HPV can be concluded to lead to very low concerns about toxicity due to its mechanism of action. In addition, it must be noted that the entire processing workflow from collection to post-processing, as demonstrated by Grossman et al. [111], which allowed healthcare workers to keep their own N95 respirators in a large academic metropolitan healthcare system, was accomplished in less than 24 h. Bailey et al. [64] validated the decontamination of a specialist transport system for patients with high-consequence infectious diseases, “EpiShuttle” (a patient transport system designed to fit into air ambulance), using HPV fumigation. The authors [64], upon decontamination with HPV, achieved a complete kill of all pathogens with the use of a commercially available Bioquell HPV system, achieving a 6 log_10_ reduction in *Geobacillus stearothermophilus* ATCC 12980 endospores, alongside organic liquid suspensions and dried surface samples of MS2 bacteriophage. However, it is important to note that HPV fumigation can be less efficient if larger amounts of biological fluids are present on the surface due to its limited penetration. Hence, a follow-up cleaning with surface disinfectant wiping is recommended in such cases. The advantages of using HPV fumigation in comparison with manual disinfection methods include better penetration into hard-to-reach areas, no risk of cross-infection due to exposure of operators, and a reduction in deviation from the manufacturer’s instructions [64]. Such advantages suggest that an HPV system could be ideal to investigate for use in medical emergencies involving new bacteria and viruses.

## 9. Conclusions

The efficacy of HPV against a broad range of clinically relevant pathogens, alongside its clinical impact, positive environmental impact, and ease of use with no-touch disinfection methods, has been repeatedly demonstrated in multiple in situ and in vitro studies. The findings of this review highlight HPV to be very close to an ideal high-level disinfectant for use in healthcare environments due to its efficacy against a broad spectrum of organisms, good material compatibility and lack of negative environmental impact. This makes HPV a biocide of choice in healthcare environments not only for traditional surface disinfection of high-touch areas but also for the decontamination of face masks and ambulances, which are important parts of healthcare systems. The promising results of HPV being able to decontaminate N95 masks during the ongoing SARS-CoV-2 pandemic, without affecting their structural integrity and filtration efficiency, demonstrate the potential for its use in emergency situations where supply chains for single-use PPE products are severely depleted. The use of HPV for mask decontamination provides a viable alternative to address the disadvantages of limited penetration due to shadowing effects and direct exposure, which can be difficult to achieve in complex geometries such as that of masks using ultraviolet light (UV) disinfection, another highly studied no-touch disinfection method. These benefits demonstrate the potential of HPV for further development as a biocide. However, this review also identifies that whilst significant efforts have been devoted to understanding the underlying mechanism of action, additional work is required, which will aid in optimising the antimicrobial activity of HPV. The authors recommend that further research should focus on performing studies on organisms wherein simultaneous damage to all bacterial cell components is investigated and correlated.

## Figures and Tables

**Table 1:** Examples of studies demonstrating the efficacy of hydrogen peroxide vapour against clinically relevant bacteria that are commonly found in hospitals.

Microorganism	Associated Diseases/Symptoms	HPV Studies
*Escherichia coli*	Blood and urinary tract infections [13]	[71,72,80]
*Staphylococcus aureus*	Blood, skin and respiratory tract infections, septicaemia and death [81]	[19,21,62,74,82,83,84,85]
*Klebsiella pneumoniae*	Urinary tract infections, pneumonia, septicaemia and soft tissue infections [75]	[48,80]
*Klebsiella oxytoca*	Urinary tract infections and pneumonia [77]	[72]
*Pseudomonas aeruginosa*	Lung and urinary tract infections [13]	[72,78,86]
*Enterococcus faecalis*/*faecium*	Blood, skin and respiratory tract infections [13]	[48,87]
*Enterobacter cloacae*	Urinary tract infections and respiratory tract infections [88]	[79]

**Table 2:** Examples of studies demonstrating the efficacy of hydrogen peroxide vapour against clinically relevant fungi that are commonly found in hospitals.

Microorganism	Associated Diseases/Symptoms	HPV Studies
*Candida* spp.	Infections of the gastrointestinal tract, vagina and oral cavity [89]	[90,91]

**Table 3:** Examples of studies demonstrating the efficacy of hydrogen peroxide vapour against clinically relevant viruses that are commonly found in hospitals.

Microorganism	Associated Diseases/Symptoms	HPV Studies
*Influenza virus* *Avian Influenza Virus* *Influenza A (H1N1)* *Swine Influenza Virus* *(H3N2)*	Influenza [89]	[35,93,94]
